# Characterization of Foot-And-Mouth Disease Viruses (FMDVs) from Ugandan Cattle Outbreaks during 2012-2013: Evidence for Circulation of Multiple Serotypes

**DOI:** 10.1371/journal.pone.0114811

**Published:** 2015-02-09

**Authors:** Alice Namatovu, Kirsten Tjørnehøj, Graham J. Belsham, Moses T. Dhikusooka, Sabenzia N. Wekesa, Vincent B. Muwanika, Hans R. Siegismund, Chrisostom Ayebazibwe

**Affiliations:** 1 National Animal Disease Diagnostics and Epidemiology Centre, Ministry of Agriculture Animal Industry and Fisheries, P. O. Box 513, Entebbe, Uganda; 2 Department of Biotechnical and Diagnostic Sciences, College of Veterinary Medicine, Animal Resources and Biosecurity, Makerere University, P. O. Box 7062, Kampala, Uganda; 3 National Veterinary Institute, Technical University of Denmark, Lindholm, DK-4771 Kalvehave, Denmark; 4 Department of Environmental Management, College of Agricultural and Environmental Sciences, Makerere University, P. O. Box 7062, Kampala, Uganda; 5 Foot-and-Mouth Disease Laboratory, Ministry of Livestock Development, P. O. Box 18021, Embakasi, Nairobi, Kenya; 6 Department of Biology, University of Copenhagen, Copenhagen N, DK-2200, Denmark

## Abstract

To investigate the foot-and-mouth disease virus (FMDV) serotypes circulating in Uganda’s cattle population, both serological and virological analyses of samples from outbreaks that occurred during 2012–2013 were performed. Altogether, 79 sera and 60 oropharyngeal fluid (OP)/tissue/oral swab samples were collected from herds with reported FMD outbreaks in seven different Ugandan districts. Overall, 61/79 (77%) of the cattle sera were positive for antibodies against FMDV by PrioCHECK FMDV NS ELISA and solid phase blocking ELISA detected titres ≥ 80 for serotypes O, SAT 1, SAT 2 and SAT 3 in 41, 45, 30 and 45 of these 61 seropositive samples, respectively. Virus neutralisation tests detected the highest levels of neutralising antibodies (titres ≥ 45) against serotype O in the herds from Kween and Rakai districts, against SAT 1 in the herd from Nwoya district and against SAT 2 in the herds from Kiruhura, Isingiro and Ntungamo districts. The isolation of a SAT 2 FMDV from Isingiro was consistent with the detection of high levels of neutralising antibodies against SAT 2; sequencing (for the VP1 coding region) indicated that this virus belonged to lineage I within this serotype, like the currently used vaccine strain. From the Wakiso district 11 tissue/swab samples were collected; serotype A FMDV, genotype Africa (G-I), was isolated from the epithelial samples. This study shows that within a period of less than one year, FMD outbreaks in Uganda were caused by four different serotypes namely O, A, SAT 1 and SAT 2. Therefore, to enhance the control of FMD in Uganda, there is need for efficient and timely determination of outbreak virus strains/serotypes and vaccine matching. The value of incorporating serotype A antigen into the imported vaccines along with the current serotype O, SAT 1 and SAT 2 strains should be considered.

## Introduction

Foot-and-mouth disease (FMD) is a highly infectious disease of cloven hoofed animals characterized by the formation of vesicles in, and around, the mouth and on the feet [1–3]. The disease is caused by infection with FMDV (genus *Aphthovirus*, family *Picornaviridae*) which exists in seven antigenically diverse serotypes (O, A, C, Asia 1, SAT 1, SAT 2 and SAT 3) [4, 5] that cause indistinguishable clinical disease [6]. FMD is endemic in Uganda with outbreaks occurring frequently [7]; cattle can show overt clinical signs, while it is generally subclinical in small ruminants [1, 8, 9]. Although mortality is generally low, this disease causes significant economic losses through reduction in milk production, loss of draught power and loss of access to profitable international livestock and livestock product markets [10–12]. Thus, control of this disease holds the potential to enhance food security, poverty alleviation and national development [11, 13].

In Uganda, control strategies for FMD outbreaks include quarantine and ring vaccination of cattle using imported trivalent vaccines (O, SAT 1 and SAT 2) [14]. However, the success of these efforts is hampered by uncontrolled animal movements, inadequate surveillance and delayed reporting of FMD outbreaks. Since vaccination against one of the seven FMDV serotypes does not protect against other serotypes [15], it is important to know which serotypes are circulating. Moreover, variation between FMDV strains within a given serotype may result in poor coverage and may necessitate matching of one or more vaccine strains against the circulating FMDVs [16], which is still a challenge in East Africa [17].

According to Vosloo et al.[6], all FMDV serotypes, other than Asia 1, have been detected in East Africa, however, serotype C has not been isolated since 2004 [18, 19]. In Uganda, the first FMD outbreak in cattle was reported in 1953 [7], with serotype O being responsible for the majority of the subsequent outbreaks. According to recent studies on Ugandan outbreaks from 2006 to 2011, topotype EA-2 serotype O FMDVs have been isolated, while the current O serotype vaccine strain incorporated in the imported trivalent vaccines belongs to the EA-1 topotype [20–22]. Other than serotype O FMDV, serotype A and SAT 2 viruses have been identified in cattle in 2002 and 2004, respectively [14, 23], while serotypes SAT 1, SAT 2 and SAT 3 FMDVs have been reported in Ugandan African buffalo (*Syncerus caffer*) [24, 25]. Very recent characterization of Ugandan and Kenyan FMDV outbreak strains disclosed simultaneous outbreaks with different strains of serotype O [22, 26] and separately SAT 2 viruses [27], emphasizing the necessity for prompt and accurate diagnosis, including regular typing of circulating strains, for effective control measures to be implemented [28].

Uganda is currently at stage 1 of the FAO/OIE defined Progressive Control Pathway for FMD (PCP-FMD) [17, 28] and to progress along this pathway, towards improved control of FMD, it is important to generate more knowledge about the epidemiology of FMD in the country. The aim of this study was to characterize the FMDVs responsible for seven of the Ugandan 2012–2013 FMD outbreaks in cattle.

## Materials and Methods

### Study area, sampling strategy and sample collection

**Figure 1:**
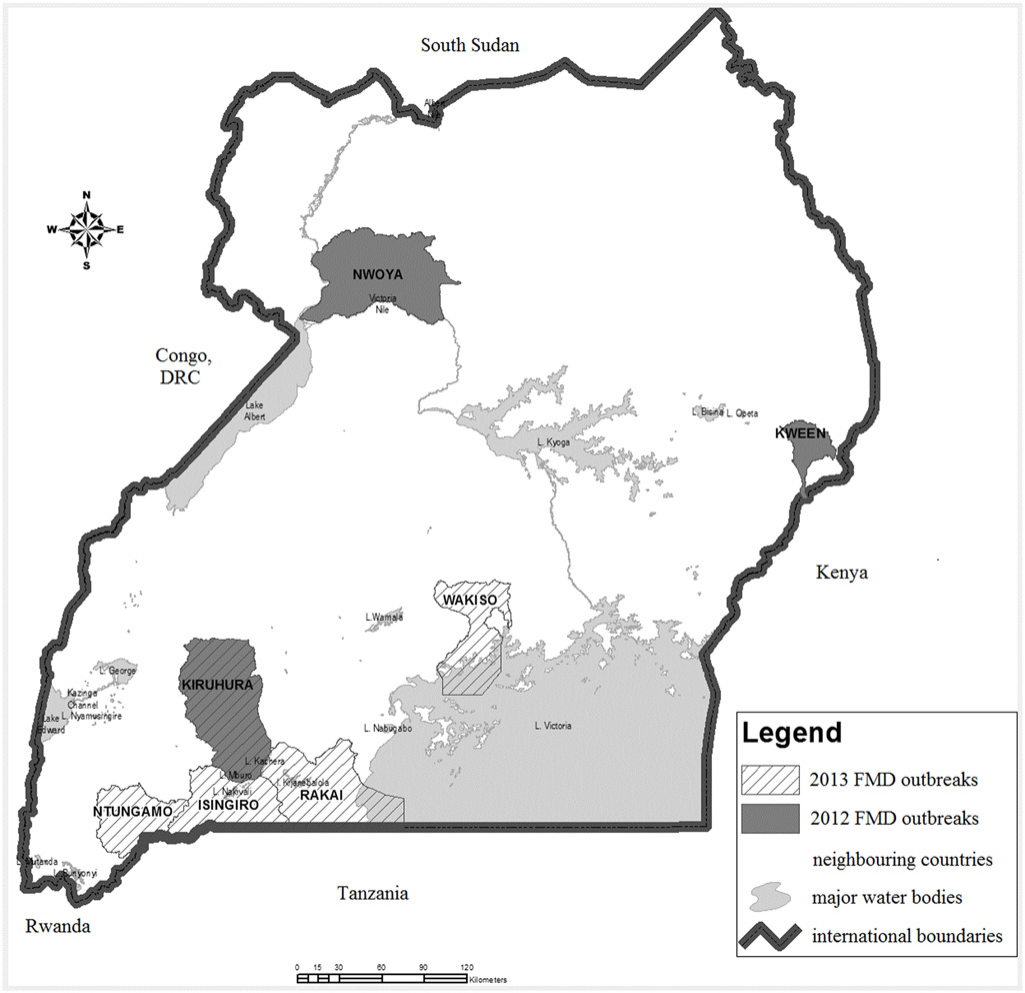
The location of reported FMD outbreaks within Uganda in 2012–2013. The map shows the seven districts of Uganda that reported FMD outbreaks during 2012–2013. These districts were: Kiruhura, Kween and Nwoya during the 2012 outbreaks plus Isingiro, Ntungamo, Rakai and Wakiso during the 2013 outbreaks.

This study was performed using outbreak samples collected from Ugandan districts with reported FMD outbreaks in 2012–2013. In 2012, 33 cattle sera were submitted to the National Animal Disease Diagnostic and Epidemiological Centre (NADDEC) from three districts (Kiruhura, Kween and Nwoya) (Fig. 1). In 2013, oral epithelial tissues, oral swabs and/or oropharyngeal fluids (OPs) were collected from one outbreak herd in each of Isingiro, Ntungamo, Rakai and Wakiso districts and in addition sera were collected from these herds in Isingiro, Ntungamo and Rakai, totaling 46 sera, 30 OPs, 16 epithelial tissues and 14 oral swabs. These samples were collected following requests by the respective District Veterinary Officers (DVOs) and subsequent approval by the Commissioner Animal Health, Ministry of Agriculture Animal Industry and Fisheries. The DVOs sought the consent of each herd owner before sampling, and samples were collected from, Isingiro District (GPS: 36M 0248868, UTM 9918082), Ntungamo District (GPS: 36M 019262, UTM 989192), Rakai District (GPS: 36M 0315178, UTM 9894397) and Wakiso District (GPS: 36N 0447204, UTM 0041487). No GPS coordinates are available for the samples submitted in 2012.The farmers were interviewed about the suspected source of the outbreak, previous outbreaks, last vaccination and type of vaccine used, while the DVOs were interviewed about the history of outbreaks and vaccination, the vaccine used and the instituted control measures.

The epithelial tissues were preserved in 50% phosphate buffered saline (PBS) and 50% glycerol while the oral swabs and OPs were preserved using PBS as recommended by OIE [16]. Sera were extracted from blood samples and stored at -20°C at NADDEC, while epithelial tissues, OPs and oral swabs were kept in liquid nitrogen during transportation to NADDEC and then stored at -80°C.

### Laboratory methods

**Detection of antibodies against FMDV non-structural proteins (NSPs)**. Sera were screened for antibodies against FMDV NSPs using the PrioCHECK FMDV NS kit (Prionics AG, Zurich Switzerland) following the manufacturer’s instructions except that optical density (OD) values were measured at dual wavelengths (450 nm and 620 nm) using a Multiskan Ascent spectrophotometer (Thermo Labsystems Oy, Helsinki, Finland). Results were expressed as percentage inhibition (PI) relative to the mean of the negative controls; PI = 100—((OD of test sample (OD_450_—OD_620_)/OD of mean negative control (OD_450_—OD_620_)) ×100), and sera with PI ≥ 50% were considered positive.

**Detection of serotype-specific antibodies against FMDV using solid phase blocking ELISAs (SPBE)**. All samples that were positive in the Priocheck FMDV NS ELISA were tested for antibodies against each of the seven FMDV serotypes using SPBEs as described [8]. Screening was done using a sample dilution of 1:10 and the results expressed as optical density percentage (ODP) using the formula; ODP = (OD of test sample (OD_450nm–620nm_) / mean OD of negative controls (OD_450nm–620nm_)) × 100. Samples were regarded as positive if ODP values were equal to or lower than: 50% for serotypes O, SAT 1, SAT 2 and SAT 3, 45% for serotype A and 35% for serotypes C and Asia 1 [8].

Positive reactions were titrated in the appropriate SPBEs (using two-fold dilution series from 1:10 to 1:1280). Titres were expressed as the reciprocal of the highest positive dilution and titres ≥ 80 were considered positive for each serotype [22].

**Detection of serotype-specific neutralising antibodies against FMDV using virus neutralization tests (VNTs)**. For each district, selected, representative samples with titres ≥ 80 in the SPBEs were tested in the corresponding VNTs for the presence of neutralising antibodies. The protocol was adapted from the procedure described in the OIE terrestrial manual [16] and was described previously [22]. Briefly, two-fold serum dilutions were reacted for 1 hour with 100 TCID_50_ of virus suspension (O Manisa, A Iraq 24/64, C Noville, Asia 1 IND 8/79, SAT 1 BOT/68, SAT 2 ZIM 5/81 or SAT 3 ZIM 4/81) in equal volumes, in 96-well plates. Subsequently, swine kidney cell suspension was added to the wells and the plates were incubated at 37°C for three days. The plates were examined for cytopathic effect (CPE) under the microscope and the 50% end-point titres were calculated according to Reed and Muench, [29]. A titre ≥ 45 was considered positive, 16 – 44 was considered doubtful and < 16 negative.

**Detection of FMDV by virus isolation (VI)**. Epithelial tissue preparation and VI procedures were adapted from the description in the OIE terrestrial manual [16] as described [25]. Briefly, samples (OPs or oral swab suspension or 10% suspension of epithelial tissues in Eagles minimal essential medium including 2 million I.U. benzylpenicillin, 1g dihydro-streptomycin sulphate, 0.5g neomycin sulphate and 8.5¼g amphotericin per litre (MEM+)) were inoculated onto monolayers of primary bovine thyroid (BTY) cells in five wells using a dilution of 1:2 and in five wells using a dilution of 1:4 in a 96 well tissue culture plate. Epithelial samples were cultured on separate plates, while for OPs/oral swabs, samples from the same herd were cultured on the same plate but separated by wells with uninfected cells. The plates were incubated for 2 hours at 37°C (with 5% CO_2_), followed by a wash with MEM+ including 2% fetal calf serum (FCS) and addition of 150μl MEM+ with 2% FCS per well. The plates were incubated for 1–2 days, as above, and observed for CPE every 24 hours. CPE-positive samples were frozen for harvesting, while samples without CPE after 48 hours were harvested by freeze-thawing and passaged again on BTY monolayers as described above. All samples that did not show CPE following the second passage were considered negative.

**Confirmation of FMDV in CPE-positive samples by antigen ELISA (Ag ELISA)**. CPE-positive harvests from all districts were tested in the Lindholm in-house antigen ELISA following a protocol based on the description in the OIE terrestrial manual [16] as described [25]. Samples giving ODs > 0.2 higher than the negative control were considered positive, while those with ODs 0.1—0.2 above the negative control were inconclusive and those with ODs < 0.1 above the negative control were considered negative.

**FMDV RNA extraction and detection using quantitative RT-PCR (RT-qPCR)**. RNA was extracted from FMDV antigen positive cell culture harvests using the Blood kit Viral RNA Mini Spin Protocol in the QIAamp Viral RNA Mini Kit (Qiagen, Hamburg, Germany). cDNA was synthesized using the Perkin Elmer (PE) Biosystem TaqMan kit (Perkin Elmer, Foster city, California, USA) and RT-qPCR was performed using the TaqMan PCR kit (PE Applied Biosystems, Foster city, California, USA) with FMDV 3D-F (ACTGGGTTTTACAAACCTGTGA) and FMDV 3D-R (GCG AGTCCTGCG ACG GA) primers and the 3D-P probe (TCCTTTGCACGCCGTGGGAC) labelled with 6-carboxyfluorescein as a reporter dye and tetramethylrhodamine as a quencher at the 5′ and 3′ ends respectively [30].

**cDNA synthesis and sequencing of FMDV VP1 coding region**. The cDNA for sequencing was synthesized using Ready-To-Go You-Prime First-strand Beads (GE Healthcare Life Sciences, Uppsala, Sweden) according to the manufacturer’s instructions using 28μl template and 7μl of a four primer mix (85 ¼l of pdN6 random hexamer (50 ng/¼l), 5 ¼l of T27V (TTTTTTTTTTTTTTTTTTTTTTTTTTTV) (25 pmol/¼l), 5 ¼l of KDS 1R (CCAGTCCCCTTCTCAGATC) (25 pmol/¼l) and 5 ¼l of 9-G PN 55 (GGGGGGGGGGGGGGH) (25 pmol/¼l)). The PCR products corresponding to the VP1-coding region were prepared for sequencing as previously described [20] except that the SAT 2 primers used were the forward 13-K PN 100 (5′–3′ GGGTGGBBGTSTWMCAGRTSACMGACAC) and reverse 1-O PN 15 (5′–3′ GAAGGGCCCAGGGTTGGACTC) (where B is C or G or T; S is G or C, W is A or T, M is A or C, R is A or G). For serotype A samples, the primers used were forward 13-K PN 101 (5′–3′ CACTGCTAYCAYKCNGARTGGGA) and reverse 10-P PN 34 (5′–3′ CAGGGTTGGACTCMACGTCTCC) (where M is A or C; Y is T or C; K is G or T; R is G or A and N is G or A or T or C). All primers were used at a concentration of 5 pmol/μl.

Gel purification of the PCR amplicons (795–942 bp) was performed using the GeneJET PCR purification kit (Thermo Fisher Scientific). Cycle sequencing was performed with 10–15 ng of PCR product (as determined using a Nanodrop spectrophotometer (Thermo Scientific, Wilmington, DE, USA)) and the BigDye terminator v3.1 cycle sequencing kit (Applied Biosystems, Foster City California, USA) on an ABI 3700 automated DNA Sequencer (Applied Biosystems).

### Data analysis

Descriptive serological data were entered in Microsoft Excel and imported into Stata 9 (Statacorp, Lakeway Drive College Station, Texas, USA) where the analysis was performed using the median and interquartile range as a measure of central tendency and spread respectively. For molecular data, base calling of nucleotide sequences was performed using seqMan Pro software (Lasergene package, DNAstar, Inc., WI, USA). Published sequences relevant to this study were obtained using BLAST searches from NCBI (http://www.ncbi.nlm.nih.gov/)[31]. The sequences analyzed correspond to the VP1 coding region and details of the new and previously published sequences are summarised in Table 1. Multiple sequences were aligned using MUSCLE (codons) [32] incorporated in MEGA 5 [33]. A phylogenetic tree of these sequences was estimated using the Neighbor-Joining method [34]. The percentage of replicate trees in which the associated taxa clustered together in the bootstrap test (2000 replicates) is shown next to the branches [35]. The tree is drawn to scale with branch lengths in the same units as those of the evolutionary distances used to infer the phylogenetic tree. The evolutionary distances were computed using the Tamura 3-parameter method [36] and are in the units of the number of base substitutions per site. The rate variation among sites was modelled with a gamma distribution (shape parameter = 1). The analysis for serotype A and SAT 2 involved 24 and 17 different nucleotide (nt) sequences, respectively, and codon positions included were 1^st^+2^nd^+3^rd^. There were a total of 638 and 648 nt positions in the final dataset for serotype A and SAT 2, respectively. Evolutionary analyses were conducted in MEGA 5 [33].

**Table 1:** Eastern Africa serotype A and SAT 2 FMDV sequences used for comparisons in this study.

**Sequence**	**Collection date**	**District/ Country**	**Accession number**	**Serotype**	**Genotype/ lineage**	**Reference**
U74/13*	26/04/2013	Wakiso, Uganda	KP089984	A	G-I	This study
U75/13*	26/04/2013	Wakiso, Uganda	KP089985	A	G-I	This study
K3/2013	2013	Thika, Kenya	KJ440876	A	G-I	[39]
K148/2012	2012	Nakuru, Kenya	KJ440874	A	G-I	[39]
K63/2009	2009	Narok, Kenya	KJ440871	A	G-I	[39]
TAN/9/2009	13/06/2009	Njombe, Tanzania	KF561693	A	G-I	[50]
TAN/45/2009	06/11/2009	Iringa, Tanzania	KF561696	A	G-I	[50]
K73/2008	2008	Loitoktok, Kenya	KJ440870	A	G-I	[39]
TAN/11/2008	01/08/2008	Iringa, Tanzania	KF561690	A	G-I	[50]
A/TUR/25/2007	06/04/2007	Mus, Turkey	FJ755125	A	Iran-05	[51]
ETH/4/2007	07/12/2007	Ethiopia	FJ798150	A	G-VII	[52]
SUD/3/2006	26/04/2006	Sudan	GU566070	A	G-IV	[53]
A/TUR/12/2005	27/12/2005	Murdagi, Turkey	FJ755100	A	Iran-05	[51]
K44/2005	2005	Nakuru, Kenya	KJ440869	A	G-I	[39]
K1/1997	1997	Meru North, Kenya	KJ440865	A	G-VII	[39]
K9/1994	1994	Narok, Kenya	KJ440863	A	G-I	[39]
SUD/1/85	6/12/1984	Soba, Sudan	GU566068	A	G-IV	[53]
K67/1985	1985	Isiolo, Kenya	KJ440858	A	G-I	[39]
K49/1984	1984	Narok, Kenya	KJ440857	A	G-I	[39]
K293/1983	1983	Taita Taveta,Kenya	KJ440855	A	G-I	[39]
K48/1981	1981	Kalifi,Kenya	KJ440852	A	G-I	[39]
K35/1980**	1980	Embu, Kenya	KJ440846	A	G-VII	[39]
K5/1980**	1980	Kajiado, Kenya	KJ440848	A	G-1	[39]
UGA/13/66	1966	Uganda	KF561705	A	G-VII	[50]
U30/13*	8/2/2013	Isingiro, Uganda	KP089986	SAT 2	I	This study
U35/13*	8/2/2013	Isingiro, Uganda	KP089987	SAT 2	I	This study
U45/13*	8/2/2013	Isingiro, Uganda	KP089988	SAT 2	I	This study
U69/13*	8/2/2013	Isingiro, Uganda	KP089989	SAT 2	I	This study
U73/13*	8/2/2013	Isingiro, Uganda	KP089990	SAT 2	I	This study
K125/12*	2012	Gucha, Kenya	N/A	SAT 2	I	unpublished
EGY/13/2012	1/3/2012	Qualubia-Banha Egypt	JX570622	SAT 2	VII	[54]
EGY/9/2012	29/2/2012	El-Suiz, Egypt	JX570622	SAT 2	VII	[54]
KEN/122/2009	1/1/2009	Rongai, Kenya	JX570630	SAT 2	IV	[54]
KEN/11/2009	2009	Kiambu, Kenya	JX570628	SAT 2	IV	[54]
TAN/43/2009	2009	Makete, Tanzania	KF561727	SAT 2	IV	[50]
K70/05	2005	Nairobi, Kenya	HM623701	SAT 2	I	[24]
UGA/11/2004	2004	Kiboga, Uganda	GU323178	SAT 2	I	[26]
TAN/6/2004	2004	Arusha, Tanzania	KF561723	SAT 2	IV	[50]
TAN/9/2004	2004	Arusha, Tanzania	KF561724	SAT 2	IV	[50]
K52/84**	1984	Nakuru, Kenya	HM623685	SAT 2	I	[24]
U267/83	1983	Uganda	HM623682	SAT 2	I	[24]

*: 2012–2013 Ugandan and one 2012 Kenyan outbreak FMDV sequences included in this study

**: vaccine strain sequences

N/A: means not available.

### Ethical approval

This research is part of a larger ongoing strategic project ‘Transboundary Animal Diseases in East Africa’. Ethical approval for the project was granted by the Ministry of Agriculture Animal Industry and Fisheries, Uganda (Reference LHE 199/01).

## Results

### Detection of antibodies against FMDV NSPs

Altogether 61 of the 79 (77%) cattle sera collected from herds with reported FMD outbreaks had antibodies against the FMDV NSPs with herd prevalences ranging from 33% to 93% (Table 2).

**Table 2:** Summary of antibody analysis by PrioCHECK FMDV NS ELISA, SPBE antibody ELISAs and VNTs.

**District^a^**	No. of samples	Anti-NSP positive	No. positive in SPBE^b^ median titre (IQR)				No. positive/no. tested in VNT^c^ median titre (IQR)				Conclusion based on serology
			O	SAT 1	SAT 2	SAT 3	O	SAT 1	SAT 2	SAT 3	
**Kiruhura^d^**	13	12	8	9	11	12	1/8	2/4	4/4	1/12	SAT 2^f^
			480	640	1280	320	113	429	566	67	
			(320–1280)	(320–1280)	(320–1280)	(320–320)		(320–538)	(170–1529)		
**Kween^d^**	11	10	9	7	1	9	9/9	0/6	nd	0/9	O
			640	320	80	320	269				
			(360–1280)	(160–640)		(320–320)	(226–640)				
**Nwoya^d^**	9	3	0	3	0	2	na	1/3	na	0/1	SAT 1
				640		160		57			
				(160–1280)		(160–160)					
**Isingiro^e^**	23	20	15	16	18	17	6/15)	6/14	13/13	1/14	SAT 2
			320	640	640	320	104	48	761	113	
			(80–640)	(320–640)	(320–1280)	(80–480)	(80–226)	(48–67)	(640–905)		
**Ntungamo^e^**	13	10	3	4	9	4	2/3	1/4	7/7	1/4	SAT 2
			80	120	160	240	81	80	190	80	
			(80–320)	(80–160)	(80–480)	(120–480)	(67–95)		(160–269)		
**Rakai^e^**	10	6	6)	6	1	1	6/6	3/6	nd	0/1	O
			320	320	80	80	193	80			
			(320–640)	(320–320)			(160–320)	(67–80)			
**Totals**	79	61	41	45	40	45	24/41	13/38	25/25	3/4	

^a^: one herd was sampled in each district

^b^: samples with titres ≥ 80 were considered positive (all anti-NSP positive samples were tested in all SPBEs).

^c^: samples with titre >45 were considered positive.

^d^: sera collected during 2012 outbreaks

^e^: sera collected during 2013 outbreaks

^f^: the two samples positive in SAT 1-VNT had higher titres in SAT 2-VNT (see S1 Table)

na: not applicable because all samples were negative in the SPBE

nd: not done

### Detection of serotype-specific antibodies against FMDV using SPBE

Antibody titres ≥ 80 against one or more of all serotypes except Asia 1 were found using SPBEs in 59 of the 61 samples that had tested positive for antibodies against FMDV NSPs. Thus, 41, 7, 1, 45, 30 and 45 samples had SPBE titres ≥ 80 against serotypes O, A, C, SAT 1, SAT 2 and SAT 3, respectively (Table 2). One sample with titre 80 in SPBE for antibodies against C FMDV (from Isingiro) and four of the seven samples with titres 80–160 in SPBE for antibodies against A FMDV (from Kween) had much higher titres against two or more of serotypes O, SAT 1, SAT 2 and SAT 3. A fifth sample from Kween and two samples from Ntungamo had SPBE A titre 160 as the highest titre. Seven sera had titres ≥ 80 against only one serotype (A: 1; SAT 1: 1; SAT 2: 3; SAT 3: 2), while 52 sera had titres ≥ 80 against multiple serotypes (data for individual animals can be found in the S1 Table). An overall evaluation of SPBE titres pointed towards the outbreaks in Nwoya and Ntungamo being caused by SAT 1 and SAT 2 FMDV, respectively, while the one in Kween was most likely caused by O FMDV and the one in Rakai by either O or SAT 1 FMDV. The SPBE results were very complicated in Isingiro and Kiruhura, where very high titres against three or more of serotypes O, SAT 1, SAT 2 and SAT 3 in most sera impeded a serotype-specific diagnosis based on these assays.

### Detection of neutralising antibodies using VNTs

Serum samples with antibody titres ≥ 80 in a serotype-specific SPBE were tested in VNTs for antibodies against the same serotype if sufficient serum was available. None of the SPBE antibody titres of 80–160 against serotype C and A were confirmed in the VNTs. Furthermore, all but three of 42 samples with SPBE antibody titres ≥ 80 against serotype SAT 3 were negative or inconclusive in the corresponding VNT. However, the number of SPBE positive samples with neutralising antibodies against serotypes O, SAT 1 and SAT 2 (titres ≥ 45) were 24/41, 13/38 and 25/25, respectively (Table 2). The VNTs determined high antibody titres against serotype O FMDV in all tested sera from Kween and Rakai with lower titres against SAT 1 in some sera from Rakai, while the detection of anti-SAT 1 antibodies in the SPBE was confirmed for samples from Nwoya (Table 2). All SPBE positive sera from Ntungamo, Kiruhura and Isingiro tested in VNT had high neutralizing titres against SAT 2 FMDV with lower titres against other serotypes in some sera (Table 2). The three sera with neutralising antibodies against SAT 3 came from three different herds/districts and had higher neutralising antibody titres against at least one other serotype (O, SAT 1, SAT 2) (see S1 Table), indicating that these apparent anti-SAT 3 reactions were likely cross reactivity generated by exposure to other serotypes.

### Virus characterization by VI, Ag ELISA, RT-qPCR and sequencing

Eight of 30 OPs, six of 16 epithelial samples and two of 14 oral swabs produced CPE during one or two passages on primary BTY cells. These samples were from the districts of Isingiro (eight), Ntungamo (two), Rakai (three) and Wakiso (three). The FMDV antigen ELISA identified serotype SAT 2 FMDV antigen in cell culture harvests from one OP, three epithelia and one oral swab from Isingiro and serotype A FMDV antigen in those from three epithelia from Wakiso (data not shown). Moreover, these eight virus harvests had Ct-values < 25 in RT-qPCR assays. From these harvests, amplicons corresponding to the complete VP1 coding region were generated by RT-PCR and sequenced. The VP1 coding sequences, between 633 and 642 nucleotides in length, were determined and compared with other sequences from NCBI, Genbank, using BLAST. The Isingiro and Wakiso sequences were most closely matched to other SAT 2 and A FMDV sequences, respectively (Table 1), consistent with the Ag ELISA data on these virus isolates. Identification of SAT 2 as the serotype responsible for the outbreak in the Isingiro district was consistent with the high titres of neutralising antibodies against SAT 2 detected in the sera from this district (Table 2).

### Phylogenetic analysis of serotype A FMDVs

**Figure 2:**
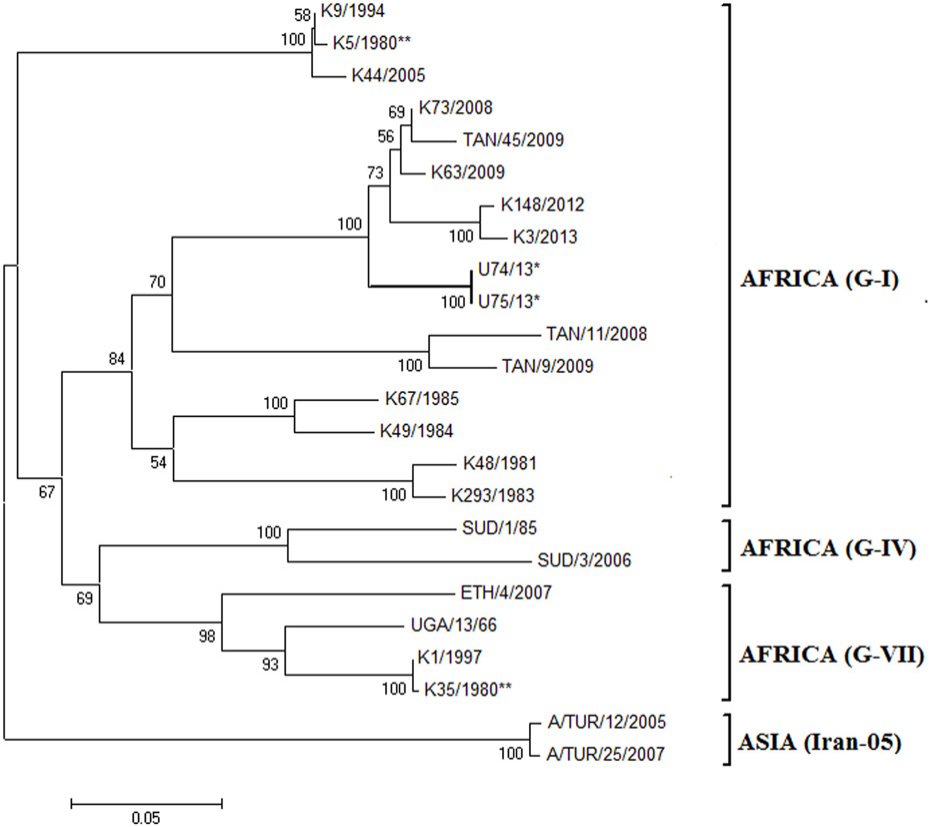
Neighbor-joining phylogeny tree based on serotype A FMDV VP1 coding sequences. The two 2013 Ugandan outbreak FMDV sequences are marked with asterisks (*), and K35/1980** and K5/1980** are the current serotype A virus vaccine strains produced in Kenya and last used in Uganda in 2002. Bootstrap values greater than 50% are marked on the tree. Two of the three global topotypes within serotype A (Africa and Asia) are indicated, as are some of the African genotypes found in Eastern Africa (G-I, G-IV and G- VII). The topotype Asia serotype A FMD viruses were used to out root the tree.

**Figure 3:**
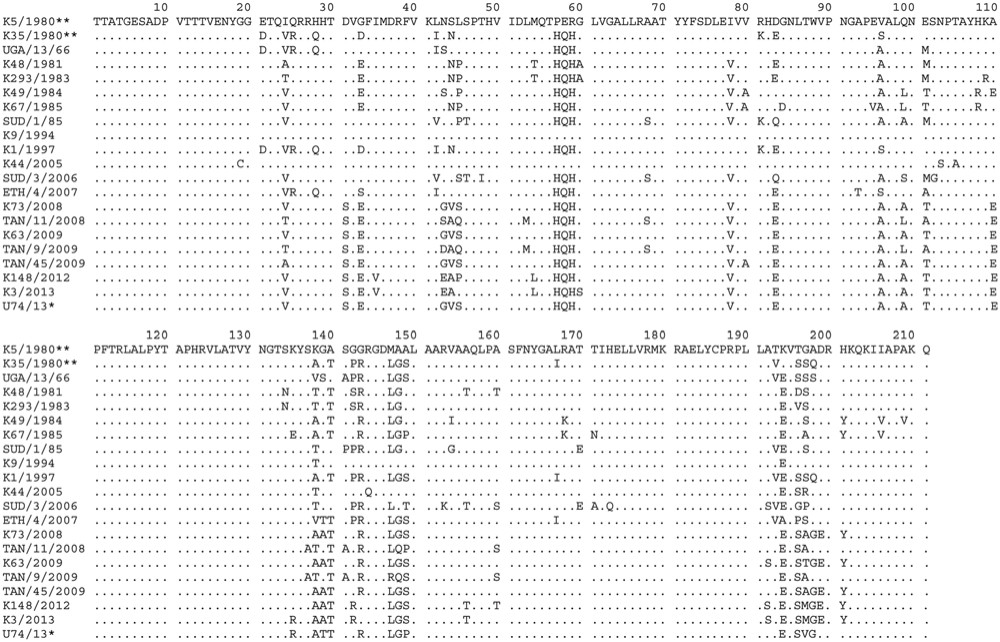
Alignment of serotype A FMDV VP1 amino acid sequences. The VP1 amino acid sequences were inferred from one of the Ugandan 2013 serotype A FMDV nucleotide sequences (marked with an asterisk (*)) and selected African serotype A FMDV strains. The dots indicate identity with the vaccine strain sequence (K5/1980**) that is the reference sequence.

Assessment of phylogenetic relationships using selected, genotype-defined serotype A FMDV isolates (Table 1) showed that the VP1 sequences of the Wakiso strains (U74/2013 and U75/2013) clustered with other strains belonging to the Africa genotype (G-I) (Fig. 2). The two Wakiso strains had 100% nt identity with each other within the VP1 coding region and so had the same predicted amino acid sequences (Fig. 3). The Wakiso viruses had pairwise nt identity of 93% with both K3/2013 and K148/12 Kenyan isolates and amino acid (aa) identity of 93% and 92% for K3/2013 and K148/12 respectively. Thus they belonged to the same lineage but were in different sub-lineages [37–39]. The Wakiso sequences had pairwise nt identity of 82% (521/633) and 82% (517/633), and pairwise aa identity of 87% and 89% (Fig. 3), with the K5/1980 (Africa (G-I)) and K35/1980 (Africa (G-VII)) vaccine strains currently produced in Kenya, respectively [40]. Hence, based on a cut off of > 15% nt difference in the VP1 coding region of FMDV (non-SAT serotypes) for separating genotypes [39, 41], these viruses belonged to different genotypes than both the current Kenyan vaccine strains.

One hundred and twelve (18%) variable sites between the Wakiso serotype A VP1 sequences and the vaccine strain (K5/1980) were determined across the 633 nucleotides, which encode substitutions at 27/211 amino acids (13%) in the deduced protein sequence (Fig. 3). The integrin receptor binding motif, RGD, (residues 144–146) within the Wakiso viruses was conserved but there were a number of amino acid substitutions in the region flanking the RGD cell attachment site (up to residues +3 and from -1 to -9). These amino acid variations occurred within known antigenic regions; for example in antigenic site 1 [42, 43] methionine (M) was replaced by leucine (L) at residue 147 while alanine (A) was replaced by glycine (G) at position 148.

### Phylogenetic assessment of SAT 2 FMDVs

**Figure 4:**
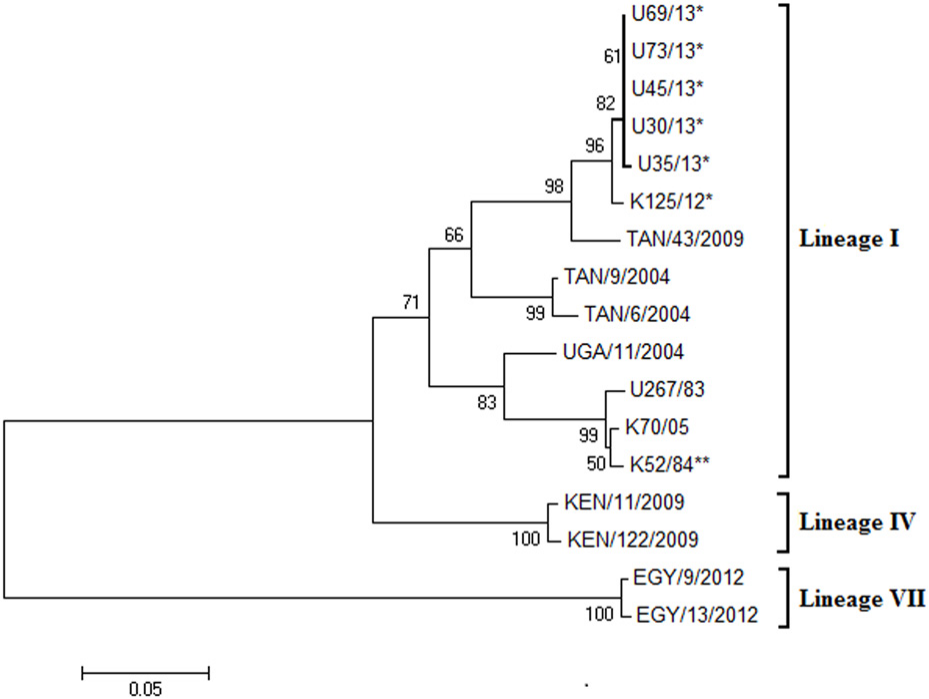
Neighbor-joining phylogeny tree based on serotype SAT 2 FMDV VP1 coding sequences. The relationships between the Ugandan 2013 SAT 2 FMD viruses and selected East African SAT 2 viruses are shown. The five 2013 Ugandan and one 2012 Kenyan outbreak FMDV (K125/12) sequences are marked with asterisks (*), while the strain K52/84** is the SAT 2 FMD virus strain incorporated in the trivalent vaccine (O, SAT 1 and SAT 2) as used in Uganda.

Except for one nucleotide substitution at position 339 (C for T) in the sequence of the virus isolate U35/13, the five Isingiro virus sequences from 2013 were identical within the VP1 coding sequence. Assessment of phylogenetic relationships with selected SAT 2 FMDV isolates (Table 1) showed that the five Isingiro viruses clustered with known strains belonging to the SAT 2 lineage I (Fig. 4). Comparison of these recent viruses with the 2004 Ugandan SAT 2 cattle isolate (UGA/11/2004), a partial VP1 sequence (345 nt), showed that they belonged to the same genotype/lineage (pairwise nt identity of 90% (309/345 nt) and aa identity of 94% (108/115 aa)) [4, 41]. However, based on the complete VP1 sequence, the Isingiro viruses were most closely related to a recent Kenyan isolate (K125/12) (Wekesa S., unpublished data) having a pairwise nt identity of 99% and aa identity of 100%, and were also closely related to a Tanzanian isolate from 2009 (TAN/43/2009) (pairwise nt identity of 97% and aa identity of 98%).

The Ugandan 2013 SAT 2 sequences had 87% nt identity (557/642 nt) and pairwise aa identity of 94% with the SAT 2 vaccine strain (K52/84) which is incorporated in the trivalent vaccines (O, SAT 1 and SAT 2) currently used in Uganda. Therefore, these viruses belonged to the same lineage as the vaccine strain.

**Figure 5:**
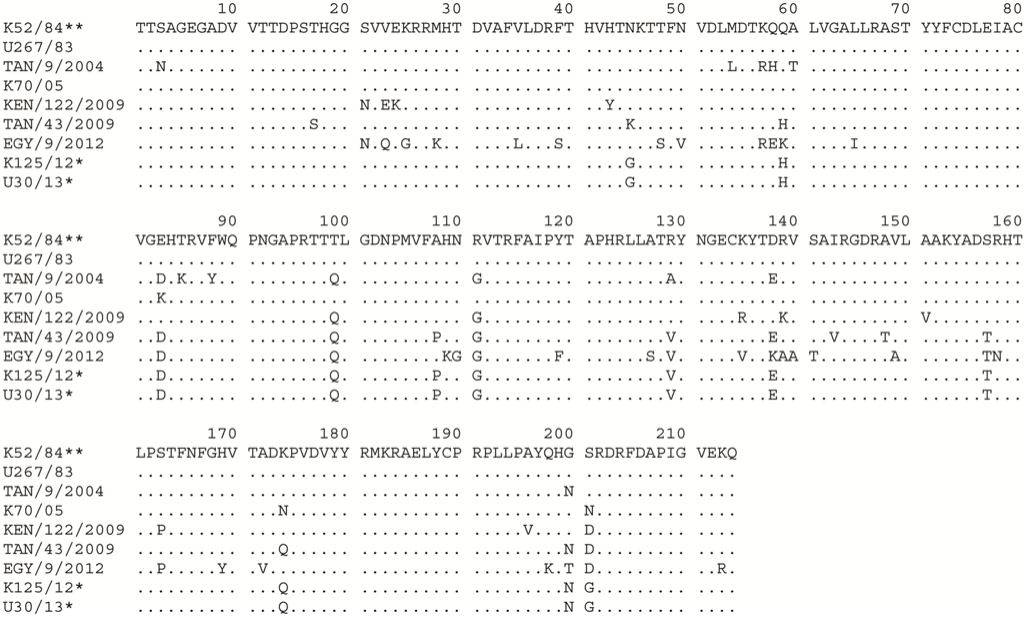
Alignment of serotype SAT 2 FMDV VP1 amino acid sequences. The VP1 amino acid sequences were inferred from one of the 2013 outbreak nucleotide sequences (marked with an asterisk (*)) and earlier SAT 2 FMDV strains. Identities with the reference vaccine strain sequence (K52/84**) are indicated by dots. Similar amino acid variation exists between the reference sequence and the Ugandan 2013 isolates and a Kenyan outbreak isolate (K125/12*) sequence (unpublished data).

Eighty five (13%) variable sites were determined across the 642 nt between the Isingiro 2013 SAT 2 outbreak sequences and the vaccine strain (K52/84) which encode substitutions of 12/214 (6%) amino acids in the deduced protein sequence (Fig. 5). The RGD motif (residues 144–146) within these viruses was conserved and so was the flanking region of the RGD motif as far as the -5 and +10 positions (Fig. 5). Conservative changes occurred at residue 138 where Asp (D) was replaced by Glu (E) and at position 157 where Ser (S) was replaced by Thr (T), both residues are located in the flanking region of the RGD motif cell attachment site [44, 45] which also constitutes an important antigenic site.

## Discussion

The aim of this study was to characterize the serotypes of FMDV responsible for Uganda’s 2012–2013 outbreaks. The detection of antibodies against FMDV NSPs in sera from herds with recent reports of clinical signs of FMD strongly supported the clinical diagnosis. Moreover, the higher seroprevalences (60–92%) of antibodies against FMDV NSPs in herds from Isingiro, Kiruhura, Kween, Ntungamo and Rakai districts probably indicate that sampling was done after considerable spread of the infection within these herds, while the lower seroprevalence in the herd in Nwoya (33%) likely indicates sampling at an earlier phase of the outbreak [9].

Determining the serotype of the causal FMDV based on the SPBEs was not straightforward, since 87% of the samples had antibody titres ≥ 80 against more than one serotype and with most herds having high titres against serotypes O, SAT 1, SAT 2 and SAT 3. In addition, the VNT also detected neutralising antibodies against more than one serotype in Ntungamo, Kiruhura and Isingiro. The cross-reactions observed between the SPBEs, and to a lesser extent between the VNTs, are in accordance with previous reports [9, 46–49], and have for the SPBEs been observed to be more prominent for the first 1–4 weeks after experimental infection of calves (Tjørnehøj, K., unpublished data). The detection of antibodies against multiple serotypes may indicate some antigenic relationship between the virus serotypes [48], or could be due to a complicated serological picture with heterotypic responses caused by priming effects from past exposures from one or more infections and/or vaccinations [9, 46, 49]. Nevertheless, overall evaluation of the SPBE titres limited the likely serotype responsible for the six outbreaks that were investigated by serology to only one serotype for three outbreaks and to just two serotypes in another outbreak. Moreover, the VNT, performed on selected SPBE positive sera, clearly identified the responsible FMDV serotype in all six outbreaks (as O, SAT 1 or SAT 2).

The serological identification of SAT 2 as the responsible virus in Isingiro was confirmed by the isolation of SAT 2 FMDV from clinical material. No serum samples were available from the Wakiso district but serotype A FMDV was successfully isolated and characterized by antigen ELISA and VP1 sequencing. Unfortunately, it was not possible to isolate viruses from the samples collected from the outbreaks in Isingiro, Ntungamo and Rakai. However, the Isingiro district borders the Kiruhura and Ntungamo districts, and since sera from these districts had the highest antibody titres against SAT 2, it is not unlikely that the outbreaks in these districts were all part of the same SAT 2 FMDV outbreak.

The 2013 outbreak of serotype A virus in the Wakiso district was close to the capital city (Kampala); this serotype has not been reported in Uganda since 2002 [14]. The population of cattle in this area is low and thus this FMD outbreak was suspected to have originated from another country, possibly via feed or fomites or newly introduced animals. The two identical (within VP1) serotype A FMDVs belonged to a different sub-lineage from those recently found in neighbouring Kenya (K148/2012 and K3/2013) (7% nt difference), hence, the Ugandan serotype A FMDV outbreak was not related to the Kenyan 2012–2013 outbreak. The VP1 sequence of the isolates had 18% diversity from both the K5/1980 (G-I) and K35/1980 (G-VII) vaccine strains. In fact, the Wakiso FMDVs cluster with viruses within a new genotype that has arisen from within the Africa topotype (G-I) as discussed recently for Kenyan FMD viruses [40].

The five SAT 2 isolates from Isingiro were collected from a single epidemiological unit and the VP1 coding sequences were identical except for one nt change in one of the five isolates and belonged to lineage (I) within the SAT 2 strains. Moreover, these isolates were only distantly related to the most recent Ugandan SAT 2 cattle isolate from 2004 (UGA/11/2004) (10% nt difference) [26], but were closely related to a recent Kenyan isolate (K125/12) (1% nt difference) and a Tanzanian isolate from 2009 (TAN43/2009) (3% nt difference). This provides further evidence for the transboundary mobility of FMDV in the East African region [20, 22], probably due to the increased, uncontrolled, cross border movement given the growth of cross border livestock trade [50]. The 2013 Ugandan SAT 2 VP1 sequences had 13% nt difference from the vaccine strain (K52/84) which showed that they were rather different although belonging to the same genotype [41].

The current results indicate resurgence of serotypes A and SAT 2 FMDV in Uganda. The significant diversity between both virus serotypes and their respective current vaccine strains included multiple amino acid variations (Figs. 3 and 5), especially in the critical antigenic regions. This indicates a need for vaccine matching studies to establish the level of protection conferred against the currently circulating viruses by the available vaccine strains.

In conclusion, the presence of antibodies against FMDV in sera collected from six unvaccinated herds following reports of FMD outbreaks supported the FMD diagnosis, and the FMDV serotypes responsible for the outbreaks were determined as O, SAT 1 and SAT 2 by the use of SPBEs and VNTs. The serology based serotyping of the outbreaks was made more complicated due to the cross-reactions identified with the SPBEs and also, to a lesser degree, with VNTs. The FMDV responsible for one of these outbreaks was confirmed by isolation of serotype SAT 2 FMDV and the FMDV involved in a seventh outbreak was identified by isolation of serotype A FMDV. The serotype A isolates were rather distantly related to the serotype A FMDV vaccine strains incorporated in the vaccines imported to Uganda in 2002 [14]. In contrast, the Ugandan 2013 SAT 2 viruses belonged to the same lineage, (I), as the FMDV vaccine strain incorporated in the trivalent vaccines (O, SAT 1 and SAT 2) imported by Uganda but have amino acid substitutions within antigenic sites. Therefore, to enhance the control of FMD in Uganda, there is need for increased surveillance, including monitoring with efficient and timely characterization of outbreak virus strains, as well as vaccine matching. The value of incorporating an appropriate serotype A FMDV vaccine strain into the imported vaccines used in Uganda should be assessed.

## Supporting Information

S1 Table Antibody analysis by PrioCHECK FMDV NS ELISA, SPBE antibody ELISAs and VNTs on individual animals. (DOCX)
