# Supplementary material for: Characterization of Foot-And-Mouth Disease Viruses (FMDVs) from Ugandan Cattle Outbreaks during 2012-2013: Evidence for Circulation of Multiple Serotypes
Source: PLoS One. 2015 Feb 9;10(2):e0114811. doi: 10.1371/journal.pone.0114811 (PMC4321839; doi:10.1371/journal.pone.0114811)
Supplement: S1 Table — (DOCX) [file pone.0114811.s001.docx]

| **Sample ID** | **NSP-ELISA results (PI)** | **SPBE titration results** | | | | | | | **VNT results** | | | | | | |
| --- | --- | --- | --- | --- | --- | --- | --- | --- | --- | --- | --- | --- | --- | --- | --- |
|  |  | **O** | **A** | **C** | **Asia 1** | **SAT 1** | **SAT 2** | **SAT 3** | **O** | **A** | **C** | **Asia 1** | **SAT 1** | **SAT 2** | **SAT 3** |
| **Isingiro district** |  |  |  |  |  |  |  |  |  |  |  |  |  |  |  |
| is.06 | 85 | - | 10 | - | - | 40 | 40 | 20 | - | * | - | - | * | * | * |
| is.23 | 92 | 20 | - | 10 | - | 40 | 320 | 40 | * | - | * | - | * | nd | * |
| is.15 | 73 | - | 10 | - | - | - | 40 | 80 | - | * | - | - | - | * | nd |
| is 22 | 70 | 80 | - | - | - | 160 | 320 | 80 | 226 | - | - | - | 14 | nd | nd |
| is.11 | 78 | 80 | - | 10 | - | 320 | 640 | 80 | 28 | - | * | - | 14 | nd | 14 |
| is.13 | 94 | 160 | - | - | - | 320 | 640 | 80 | 40 | - | - | - | 48 | 640 | 14 |
| is.09 | 53 | 40 | - | - | - | 80 | 1280 | 80 | * | - | - | - | nd | 640 | 14 |
| is.19 | 93 | >1280 | 10 | 10 | 10 | >1280 | 80 | 320 | 381 | * | * | * | 48 | nd | 14 |
| is.10 | 64 | 80 | - | - | 20 | 320 | 320 | 320 | 14 | - | - | * | 20 | 80 | 14 |
| is.08 | 89 | 160 | 10 | 10 | 10 | 320 | 640 | 320 | 24 | * | * | * | 48 | 269 | 14 |
| is.14 | 66 | 80 | 10 | - | - | 640 | 640 | 320 | 24 | * | - | - | 67 | 905 | 24 |
| is.18 | 96 | 320 | - | - | - | 640 | 640 | 320 | 17 | - | - | - | 24 | 905 | nd |
| is.07 | 83 | 320 | - | - | - | 640 | 1280 | 320 | 40 | - | - | - | 24 | 761 | 17 |
| is.05 E | 73 | 320 | 10 | 40 | - | 640 | 1280 | 320 | 34 | * | * | - | 34 | 905 | 17 |
| is.04 | 91 | 640 | 20 | - | - | >1280 | 1280 | 320 | 40 | * | - | - | 67 | 761 | 20 |
| is.01 | 88 | 320 | - | - | - | 80 | 1280 | 640 | 113 | - | - | - | nd | 905 | 14 |
| is.12 | 97 | >1280 | 40 | 80 | - | 640 | 1280 | 640 | 95 | * | 14 | - | 34 | 1280 | 40 |
| is.17 | 96 | >1280 | - | - | - | 640 | 1280 | 1280 | 57 | - | - | - | 48 | 640 | 113 |
| is.02 | 95 | 320 | - | - | 10 | >1280 | 1280 | 1280 | 80 | - | - | * | 40 | 1076 | 34 |
| is.24 | 85 | 10 | - | - | - | - | 160 | - | * | - | - | - | - | nd | - |
| is.03 | 46 |  |  |  |  |  |  |  |  |  |  |  |  |  |  |
| is.20 | 41 |  |  |  |  |  |  |  |  |  |  |  |  |  |  |
| is.16 | 17 |  |  |  |  |  |  |  |  |  |  |  |  |  |  |
| Sub total | 20/23 | 15/20 | 0/20 | 1/20 | 0/20 | 16/20 | 18/20 | 17/20 | 6/15 |  | 0/1 |  | 6/14 | 13/13 | 1/14 |
| **Kiruhura district** |  |  |  |  |  |  |  |  |  |  |  |  |  |  |  |
| 55/se/14 | 66 | - | 10 | - | - | 40 | 40 | 320 | - | * | - | - | * | * | 14 |
| 55/se/13 | 63 | - | 20 | - | - | 40 | 80 | 320 | - | * | - | - | * | nd | 14 |
| 55/se/12 | 60 | 40 | 10 | - | - | 20 | 160 | 320 | * | * | - | - | * | nd | 14 |
| 55/se/6 | 94 | 160 | - | - | - | 160 | 320 | 320 | 17 | - | - | - | nd | nd | 14 |
| 55/se/7 | 90 | 320 | - | - | - | 640 | 320 | 320 | 28 | - | - | - | nd | nd | 20 |
| 55/se/2 | 96 | 640 | 40 | 40 | - | 320 | 640 | 320 | 14 | * | * | - | 17 | 113 | 14 |
| 55/se/4 | 89 | 320 | 10 | 10 | - | 320 | 1280 | 320 | 40 | * | * | - | nd | nd | 14 |
| 55/se/12 | 96 | 320 | - | - | - | 640 | 1280 | 320 | 14 | - | - | - | nd | nd | 14 |
| 55/se/9 | 99 | >1280 | - | - | - | 640 | 1280 | 320 | 40 | - | - | - | nd | nd | 14 |
| 55/se/3 | 83 | 1280 | - | 40 | - | >1280 | 1280 | 320 | 28 | - | * | - | 14 | 226 | 14 |
| 55/se/5 | 95 | >1280 | - | - | - | >1280 | 1280 | 320 | 113 | - | - | - | 538 | 905 | 67 |
| 55/se/8 | 92 | - | - | - | - | >1280 | 1280 | 320 | - | - | - | - | 320 | 2153 | 20 |
| 55/se/10 | -6 |  |  |  |  |  |  |  |  |  |  |  |  |  |  |
| Sub total | 12/13 | 8/12 | 0/12 | 0/12 | - | 9/12 | 11/12 | 12/12 | 1/8 |  |  |  | 2/4 | 4/4 | 1/12 |
| **Kween district** |  |  |  |  |  |  |  |  |  |  |  |  |  |  |  |
| kween/se/1 | 80 | 640 | 40 | - | - | 40 | 10 | 320 | 453 | * | - | - | * | * | 14 |
| kween/se/6 | 84 | 80 | - | - | - | 40 | - | 320 | 226 | - | - | - | * | - | 14 |
| kween/se/10 | 56 | 80 | - | - | - | 80 | - | 320 | 190 | - | - | - | nd | - | 14 |
| kween/se/2 | 83 | 640 | 20 | 10 | - | 160 | 10 | 320 | 269 | * | * | - | 14 | * | 20 |
| kween/se/4 | 84 | 640 | 160 | 10 | - | 160 | 10 | 320 | 640 | 20 | * | - | 24 | * | 14 |
| kween/se/7 | 80 | 640 | 80 | 10 | 10 | 320 | 20 | 320 | 269 | 24 | * | * | 17 | * | 14 |
| kween/se/8 | 91 | >1280 | 40 | 10 | - | 640 | 10 | 320 | 226 | * | * | - | 17 | * | 17 |
| kween/se/3 | 97 | >1280 | 80 | - | - | 640 | 20 | 320 | 640 | 28 | - | - | 40 | * | 17 |
| kween/se/11 | 94 | >1280 | 80 | 10 | 10 | 640 | 80 | 320 | 1280 | 28 | * | * | 24 | nd | 14 |
| kween/se/5 | 83 | - | 160 | - | - | - | - | - | - | 34 | - | - | - | - | - |
| kween/se/09 | 28 |  |  |  |  |  |  |  |  |  |  |  |  |  |  |
| Sub total | 10/11 | 9/10 | 5/10 | 0/10 | 0/10 | 7/10 | 1/10 | 9/10 | 9/9 | 0/5 |  |  | 0/6 |  | 0/9 |
| **Nwoya district** |  |  |  |  |  |  |  |  |  |  |  |  |  |  |  |
| nyoya/6 | 76 | - | - | - | - | 160 | - | 40 | - | - | - | - | 20 | - | * |
| nyoya/kabenge | 53 | - | 10 | - | - | 640 | 20 | 160 | - | * | - | - | 40 | * | nd |
| nyoya/3 | 81 | 20 | - | - | - | >1280 | 10 | 160 | * | - | - | - | 57 | * | 28 |
| nyoya/1 | 39 |  |  |  |  |  |  |  |  |  |  |  |  |  |  |
| nyoya/5 | 34 |  |  |  |  |  |  |  |  |  |  |  |  |  |  |
| nyoya/ywaya | 34 |  |  |  |  |  |  |  |  |  |  |  |  |  |  |
| nyoya/4 | 28 |  |  |  |  |  |  |  |  |  |  |  |  |  |  |
| nyoya/9 | 18 |  |  |  |  |  |  |  |  |  |  |  |  |  |  |
| nyoya/8 | 17 |  |  |  |  |  |  |  |  |  |  |  |  |  |  |
| Sub total | 3/9 | 0/3 | 0/3 | - | - | 3/3 | 0/3 | 2/3 |  |  |  |  | 1/3 |  | 0/1 |
| **Ntungamo district** |  |  |  |  |  |  |  |  |  |  |  |  |  |  |  |
| Nt/f2/11 | 85 | - | - | - | - | 10 | 40 | 10 | - | - | - | - | * | * | * |
| Nt/f2/01 | 78 | - | 160 | - | - | 40 | 80 | 20 | - | 14 | - | - | * | 160 | * |
| Nt/f2/03 | 74 | 10 | - | - | - | 40 | 80 | 20 | * | - | - | - | * | nd | * |
| Nt/f2/12 | 91 | - | 160 | - | - | 40 | 80 | 40 | - | 17 | - | - | * | nd | * |
| Nt/f2/04 | 84 | - | - | - | - | 80 | 320 | 40 | - | - | - | - | 17 | 113 | * |
| Nt/f2/06 | 75 | - | - | - | - | 160 | 160 | 80 | - | - | - | - | 80 | 269 | 14 |
| Nt/f2/05 | 60 | 80 | 20 | - | - | 40 | 160 | 160 | 95 | * | - | - | * | 190 | 28 |
| Nt/f1/01 | 58 | 40 | - | - | - | 80 | 80 | 320 | * | - | - | - | 14 | 269 | 24 |
| Nt/f2/02 | 81 | 320 | - | 10 | - | 160 | 640 | 640 | 67 | - | * | - | 20 | 380 | 80 |
| Nt/f2/09 | 86 | 80 | - | - | - | - | 1280 | - | 14 | - | - | - | - | 190 | - |
| Nt/f2/07 | 30 |  |  |  |  |  |  |  |  |  |  |  |  |  |  |
| Nt/f2/08 | 29 |  |  |  |  |  |  |  |  |  |  |  |  |  |  |
| Nt/f2/10 | 25 |  |  |  |  |  |  |  |  |  |  |  |  |  |  |
| Sub total | 10/13 | 3/10 | 2/10 | 0/10 | - | 4/10 | 9/10 | 4/10 | 2/3 | 0/2 |  |  | 1/4 | 7/7 | 1/4 |
| **Rakai district** |  |  |  |  |  |  |  |  |  |  |  |  |  |  |  |
| rak/f1/08 | 84 | 160 | 10 | 10 | - | 320 | 10 | 40 | 67 | * | * | - | 67 | * | * |
| Rak/f1/1 | 70 | 320 | 10 | - | - | 320 | 10 | 20 | 160 | * | - | - | 80 | * | * |
| Rak/f1/05 | 93 | 320 | - | - | - | 320 | 20 | 20 | 226 | - | - | - | 40 | * | * |
| Rak/f1/06 | 94 | 640 | 40 | 10 | 40 | 320 | 10 | 40 | 320 | * | * | * | 20 | * | * |
| rak/f1/07 | 50 | 640 | 10 | 10 | - | 320 | 80 | 80 | 320 | * | * | - | 14 | nd | 14 |
| rak/f1/10 | 60 | 320 | - | - | 10 | 640 | 20 | 40 | 160 | - | - | * | 80 | * | * |
| Rak/f1/02 | 19 |  |  |  |  |  |  |  |  |  |  |  |  |  |  |
| rak/f1/09 | 19 |  |  |  |  |  |  |  |  |  |  |  |  |  |  |
| rak/f1/04 | 3 |  |  |  |  |  |  |  |  |  |  |  |  |  |  |
| rak/f1/03 | -3 |  |  |  |  |  |  |  |  |  |  |  |  |  |  |
| subtotal | 6/10 | 6/6 | 0/6 | 0/6 | 0/6 | 6/6 | 1/6 | 1/6 | 6/6 |  |  |  | 3/6 |  | 0/1 |

-:samples negative on screening with SPBEs; *:samples negative after titration in the respective SPBEs and nd: samples not run in the respective VNTs due to insufficient sample volumes.
